# COVID-19-associated acute kidney injury patients treated with renal replacement therapy in the intensive care unit: A multicenter study in São Paulo, Brazil

**DOI:** 10.1371/journal.pone.0261958

**Published:** 2022-01-14

**Authors:** Farid Samaan, Elisa Carneiro de Paula, Fabrizzio Batista Guimarães de Lima Souza, Luiz Fernando Cardoso Mendes, Paula Regina Gan Rossi, Rafaela Andrade Penalva Freitas, Fernando Takahashi Nakagawa, Alexandre Toledo Maciel, Sylvia Aranha, Eduardo Osawa, Henrique Pinheiro Konigsfeld, Riberto Garcia da Silva, Ricardo Barbosa Cintra de Souza, Saurus Mayer Coutinho, Tales Dantas Vieira, Karina De Bonis Thomaz, Elias Marcos Silva Flato, Renata Cristina da Silva, Lucas Vicente Andrade, Muna Badaoui, Eduardo Pogetti Badaoui, Miguel Ângelo Goes, Sergio Henrique do Amaral, Karlla Cunha, Inês Marin Muniz, Jacqueline Siqueira Sampaio, Marcelino de Souza Durão Junior, Dirce M. Trevisan Zanetta, Emmanuel A. Burdmann

**Affiliations:** 1 Department of High Complexity Patients, Grupo NotreDame Intermédica, São Paulo, Brazil; 2 Nephrology Division, University of São Paulo Medical School, São Paulo, Brazil; 3 Nephrology Division, Federal University of São Paulo, São Paulo, Brazil; 4 Planning and Evaluation Group, São Paulo State Health Department, São Paulo, Brazil; 5 Nephrology Division, Leforte Liberdade Hospital, São Paulo, Brazil; 6 Medical Board, Municipal Hospital Vereador José Storopolli, São Paulo, Brazil; 7 Internal medicine, Dante Pazzanese Institute of Cardiology, São Paulo, Brazil; 8 Imed Research Group, São Camilo Pompeia Hospital, São Paulo, Brazil; 9 Nephrology Division, Santa Cruz Hospital, São Paulo, Brazil; 10 Nephrology Division, Sepaco Hospital, São Paulo, Brazil; 11 Nephrology Division, Cruzeiro do Sul Hospital, Osasco, São Paulo, Brazil; 12 Nephrology Division, São Francisco Hospital, Cotia, São Paulo, Brazil; 13 Department of General Surgery, Ipiranga Hospital Care Management Unit, São Paulo, Brazil; 14 Nephrology Division, Vila Nova Brasilândia Hospital, São Paulo, Brazil; 15 Medical Board, Bosque da Saúde Hospital, São Paulo, Brazil; 16 Medical Board, Intermédica Guarulhos Hospital, Guarulhos, São Paulo, Brazil; 17 Nephrology Division, São Paulo Hospital, São Paulo, Brazil; 18 School of Public Health, University of São Paulo, São Paulo, Brazil; University of KwaZulu-Natal, SOUTH AFRICA

## Abstract

**Introduction:**

Multicenter studies involving patients with acute kidney injury (AKI) associated with the disease caused by the new coronavirus (COVID-19) and treated with renal replacement therapy (RRT) in developing countries are scarce. The objectives of this study were to evaluate the demographic profile, clinical picture, risk factors for mortality, and outcomes of critically ill patients with AKI requiring dialysis (AKI-RRT) and with COVID-19 in the megalopolis of São Paulo, Brazil.

**Methods:**

This multicenter, retrospective, observational study was conducted in the intensive care units of 13 public and private hospitals in the metropolitan region of the municipality of São Paulo. Patients hospitalized in an intensive care unit, aged ≥ 18 years, and treated with RRT due to COVID-19-associated AKI were included.

**Results:**

The study group consisted of 375 patients (age 64.1 years, 68.8% male). Most (62.1%) had two or more comorbidities: 68.8%, arterial hypertension; 45.3%, diabetes; 36.3%, anemia; 30.9%, obesity; 18.7%, chronic kidney disease; 15.7%, coronary artery disease; 10.4%, heart failure; and 8.5%, chronic obstructive pulmonary disease. Death occurred in 72.5% of the study population (272 patients). Among the 103 survivors, 22.3% (23 patients) were discharged on RRT. In a multiple regression analysis, the independent factors associated with death were the number of organ dysfunctions at admission and RRT efficiency.

**Conclusion:**

AKI-RRT associated with COVID-19 occurred in patients with an elevated burden of comorbidities and was associated with high mortality (72.5%). The number of organ dysfunctions during hospitalization and RRT efficiency were independent factors associated with mortality. A meaningful portion of survivors was discharged while dependent on RRT (22.3%).

## Introduction

Approximately 20 to 25% of patients with acute kidney injury (AKI) in intensive care units (ICUs) require renal replacement therapy (RRT) [1,2]. The mortality rate for patients with AKI requiring dialysis (AKI-RRT) in the ICU is greater than 50% [3–6], which can be explained by the increase in age and the burden of comorbidities in critically ill patients in recent years. The COVID-19 pandemic caused by the new coronavirus SARS-CoV-2 significantly affected nephrological practice, increasing the demand for nephrologists, nephrology-specialized nurses, RRT equipment and supplies [7]. Knowing the incidence, characteristics, and outcomes of AKI associated with COVID-19 is essential for health planning.

The frequencies of AKI and AKI-RRT reported in patients with COVID-19 range from 10 to 17% and 2.4 to 4.3%, respectively [8,9]. Subgroup analyses showed a higher odds ratio (OR) for AKI in the elderly population (OR = 3.53, CI 2.92–4.25; p <0.001) and in males (OR = 1.36, CI 0.84–2.20; p = 0.21) [9]. Patients with AKI associated with COVID-19 are 15 times more likely to die [10,11], and this increase is proportional to the stage of AKI (4.5, 8.0, 19.9, and 30.2 times more likely in stages 1, 2, 3—no dialysis, and 3—requiring dialysis, respectively, p <0.001) [12].

The available information on the mortality and recovery of patients with AKI-RRT associated with COVID-19 is mostly from cohorts in high-income countries located in the Northern Hemisphere, which show a mortality rate of 63.3% to 79.3% and dependence on RRT after discharge in 22.0% to 38% of survivors [13–16]. Data on the epidemiological profile and outcomes of patients with AKI-RRT associated with SARS-CoV-2 are scarce in Latin American countries.

The objectives of this study were to evaluate the demographic profile, clinical picture, risk factors for mortality, and outcomes of critically ill patients with AKI-RRT-associated COVID-19 in the megalopolis of São Paulo, Brazil.

## Patients and methods

### Study design, location, and population

This was a multicenter, retrospective, observational study conducted in 13 hospitals (public and private) in the metropolitan region of the city of São Paulo. The choice of participating centers was made by convenience.

The inclusion criteria were as follows: patients ≥ 18 years of age admitted to the ICU due to COVID-19 between 1 April 2020 and 31 August 2020 who required RRT due to AKI. The exclusion criteria were patients with chronic kidney disease (CKD) dependent on RRT before hospitalization and those in exclusive palliative care. The study was approved by the research ethics committees of the participating centers under CAAE (certificate of presentation of ethical appreciation) number n31693820.8.1001.5485.

### Variables of interest

Demographic data (age, sex, and ethnicity), profiles of comorbidities, and hospital admission parameters (symptoms and time of symptom onset related to COVID-19, vital signs, and laboratory tests) were obtained. A patient was considered to have hypertension (HTN) if the diagnosis was recorded in the medical record, or the patient used antihypertensive drugs. A patient was considered to have diabetes mellitus (DM) if the diagnosis was recorded in the medical record, or the patient reported the previous use of oral antidiabetics drugs or insulin. A patient was considered to have CKD, heart failure (HF), chronic liver disease, and chronic obstructive pulmonary disease (COPD) if such diagnoses were reported in the medical record. A patient was considered to have coronary artery disease if she or he had a history of acute myocardial infarction, stent placement, or myocardial revascularization. A patient was considered obese with a body mass index > 30 kg/m^2^ or if this diagnosis was reported in the medical record. A patient was considered to have anemia if the serum hemoglobin (Hb) concentration at admission was < 13.0 g/dl in men or Hb < 12.0 g/dl in women. In addition, the following variables were of interest: the severity of pulmonary involvement on chest tomography (mild, < 25%; moderate, between 25 and 50%; and severe, > 50%), Simplified Acute Physiology Score (SAPS) at ICU admission, the incidence of organ dysfunction other than kidney dysfunction during hospitalization (pulmonary, circulatory, hepatic, or coagulation) and the use of the following medications during hospitalization: azithromycin, hydroxychloroquine, corticosteroids, antibiotics (in addition to azithromycin), and heparin by continuous infusion. Pulmonary, circulatory, hepatic, and coagulation dysfunction was defined as a PaO_2_/FiO_2_ ratio < 400 or the need for mechanical ventilation, the use of vasopressors, serum levels of total bilirubin ≥ 1.2 mg/dl, and platelets < 150,000/mm^3^, respectively [17].

The RRT parameters evaluated were serum creatinine, urea, sodium, potassium, and bicarbonate up to 24 hours before the first RRT session, simple mean values of serum creatinine, urea, sodium, potassium, and bicarbonate during the period on RRT and the RRT method used: peritoneal dialysis (PD), intermittent hemodialysis (IHD), sustained low-efficiency dialysis (SLED), or continuous renal replacement therapy (CRRT). Efficient RRT was defined as the presence of two or more of the following criteria, which were evaluated during the period in which the patient underwent RRT: simple mean values of urea < 100 mg/dl, potassium < 5.0 mEq/l, and bicarbonate > 22 mEq/l. The number of RRT sessions, the total hours of CRRT, and the indications for the RRT method could not be obtained.

The outcomes of interest were the length of hospital stay, death, and discharge (with or without RRT dependence). The patient follow-up time was up to 90 days of hospitalization.

The diagnosis of COVID-19 was defined as a positive RT–PCR (real-time polymerase chain reaction) result or as a combination of respiratory symptoms and chest tomography with typical changes (peripheral and bilateral ground glass opacities, multifocal ground glass opacities of rounded morphology, and/or an inverted halo sign) [18].

### Statistical analysis

Categorical variables are presented as frequencies. Quantitative variables with a normal distribution are presented as the mean and standard deviation, and those with a no normal distribution are presented as the median and interquartile range. Frequencies were compared using the χ^2^ or Fischer’s test as appropriate. Intergroup comparisons for quantitative variables were performed using Student’s t test and the Mann–Whitney U test for normally distributed and nonnormally distributed data, respectively.

The analysis of independent risk factors for mortality was performed using logistic regression. Due to the occurrence of missing data and their no monotonic pattern, multiple inputs were performed using the Monte Carlo method via Markov chains, which generated 15 complete datasets. All variables included in the multiple logistic regression were used for the input of missing data, for which logistic regression was performed for categorical variables and linear regression was performed for continuous variables. Multiple logistic regression analysis was performed for each of the 15 complete datasets using a backward strategy and maintaining in the model the variables that, when removed, led to a change greater than 10% in the estimate of the betas of the variables present. The presence of multicollinearity was verified by the variance inflation factor.

The initial logistic regression model included the following variables: type of hospital (public or private), age, sex, smoking, one or more comorbidities (no reference), lower tercile of platelets, upper tercile of creatinine at admission, number of organ dysfunctions during hospitalization (circulatory, pulmonary, coagulopathic, and hepatic), use of vasoactive drugs, mechanical ventilation, urea > 150 mg/dl, potassium > 5 mEq/l, bicarbonate < 22 mEq/l, upper tercile of serum creatinine levels measured on the day of the first indication for RRT, and efficient RRT. The quality of the model fit was evaluated using the Hosmer–Lemeshow test, and the significance of the variables was evaluated using the Wald test. The results obtained with the analysis of the 15 datasets were averaged [19]. Statistical analysis was performed using SPSS software, version 19.0 (SPSS Inc., Chicago, IL, USA). The significance level adopted was < 0.05.

## Results

**Fig 1:**
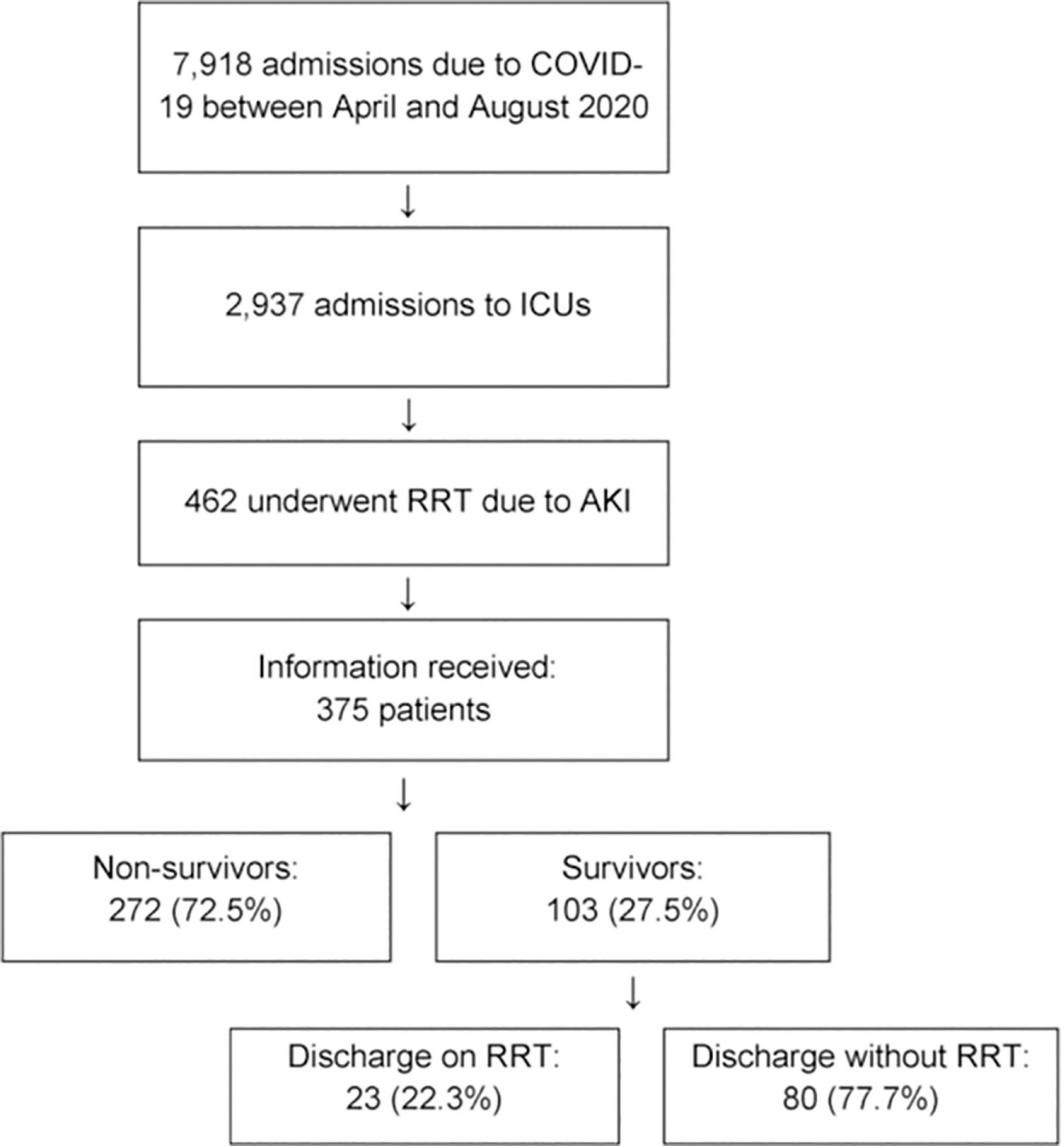
Patient inclusion flowchart and outcomes. COVID-19, a disease caused by the new coronavirus; ICUs: Intensive care units; RRT, renal replacement therapy; AKI, acute kidney injury.

Between April and August 2020, the numbers of patients—total and ICU—admitted for COVID-19 in the participating hospitals were 7,918 and 2,937, respectively. In the same period, 462 of these 2,937 ICU patients (15.7%) underwent RRT due to AKI. The data for 375 (81.1%) of these patients (39.5% admitted to public hospitals and 60.5% admitted to private hospitals) were obtained (Fig 1). Patients with positive RT–PCR results for COVID-19 accounted for 97.1% of the population (364 patients). Individuals with unavailable RT–PCR results but with clinical and radiological findings highly compatible with COVID-19 accounted for 2.9% of the population (11 patients).

The demographic data, comorbidities, and hospital admission parameters are summarized in Table 1. The age was 64.1 (55.0–74.2) years, and a predominance of males (68.8%) and white ethnicity (38.9%) was noted. The main comorbidities were HTN (68.8%), DM (45.3%), anemia (36.3%), obesity (30.9%), CKD (18.7%), coronary artery disease (15.7%), and HF (10.4%). Among the patients, 62.1% had two or more comorbidities.

**Table 1:** General characteristics of the study population (N = 375).

Demographics	
Age, years	64.1 (55.0–74.2)
Male sex, % (n)	68.8 (258)
Ethnicity	
White, % (n)	38.9 (146)
Afro-descendants, % (n)	19.0 (71)
Asian, % (n)	3.7 (14)
Missing data, % (n)	38.1 (143)
Smoking, % (n)	18.1 (68)
Comorbidities	
Hypertension, % (n)	68.0 (255)
Diabetes mellitus, % (n)	45.3 (170)
Obesity	
Yes, % (n)	30.9 (116)
No, % (n)	46.9 (176)
Unknown, % (n)	22.1 (83)
Chronic kidney disease, % (n)	18.7 (70)
Coronary insufficiency, % (n)	15.7 (59)
Heart failure, % (n)	10.4 (39)
Chronic obstructive pulmonary disease, % (n)	8.5 (32)
Neoplasia, % (n)	3.5 (13)
Chronic liver disease, % (n)	2.1 (8)
Participating institution	
Public hospital, % (n)	39.5 (148)
Private hospital, % (n)	60.5 (227)
Parameters at hospital admission	
Symptoms	
Dyspnea, % (n)	74.4 (279)
Cough, % (n)	74.1 (278)
Fever, % (n)	52.3 (196)
Diarrhea, % (n)	10.9 (41)
Coryza, % (n)	9.3 (35)
Odynophagia, % (n)	6.9 (26)
Expectoration, % (n)	6.7 (25)
Anosmia, % (n)	6.7 (25)
Ageusia, % (n)	4.8 (18)
Mean arterial pressure (mmHg)	91.3 ± 18.5
Oxygen saturation (%)	92 (88–95)
Time since symptom onset, days	5 (3–7)

Data are presented as the mean ± SD, the median and interquartile range (p25-p75), or a percentage.

Subsidiary examinations, clinical characteristics, organ dysfunction, treatments, and outcomes of the patients are shown in Table 2. Severe pulmonary involvement occurred in 39.7% of the patients (chest tomography was not performed or not reported for 15.2%). Organ dysfunction, in addition to kidney dysfunction, included circulatory (85.3%), pulmonary (78.7%), coagulopathic (30.4%), and hepatic dysfunction (13.3%). The isolated use of IHD, SLED, and CRRT was 56.5% (212), 8.3% (31), and 18.7% (70), respectively. A total of 16.5% (62) of patients received any combination of RRT methods. No patient underwent PD. Death occurred in 72.5% of the population (272/375 patients). Among the survivors, 22.3% (23/103 patients) were discharged while dependent on RRT.

**Table 2:** Subsidiary test results, clinical characteristics, and outcomes of the patients included in the study (N = 375).

Hospital admission exams	Normal range	
Hemoglobin (g/dl)	12.0–16.0	13.0 ± 2.1
Total leukocytes (n/mm^3^)	4,000–8,000	8,117 (5,500–11,200)
Total lymphocytes (n/mm^3^)	1,000–3,900	974 (699–1489)
Platelets (n/mm^3^)	150,000–400,000	179,000 (141,000–231,750)
Serum creatinine (mg/dl)	0.6–1.3	1.16 (0.90–1.78)
D-dimer (ng/ml)	< 0.5	1.5 (0.7–9.1)
C-reactive protein (mg/dl)	< 0.1	15.5 (7.6–25.1)
Pulmonary involvement on tomography		
Mild, % (n)		13.1 (49)
Moderate, % (n)		32.0 (120)
Severe, % (n)		39.7 (149)
Unknown, % (n)		15.2 (57)
Severity score at ICU admission		
SAPS 3		54 (46–66)
Unknown, % (n)		42.9 (161)
Mechanical ventilation, % (n)		88.5 (332)
Organ dysfunctions during hospitalization		
Circulatory, % (n)		85.3 (320)
Pulmonary, % (n)		78.7 (295)
Coagulopathy, % (n)		30.4 (114)
Hepatic		
Yes, % (n)		13.3 (50)
No, % (n)		73.3 (275)
Unknown, % (n)		13.3 (50)
Kidney, % (n)		100.0 (375)
Medications		
Azithromycin + hydroxychloroquine, % (n)		37.6 (141)
Other antibiotics, not azithromycin		
Yes, % (n)		85.9 (322)
No, % (n)		2.7 (10)
Unknown, % (n)		11.5 (43)
Corticosteroids, % (n)		60.3 (226)
Continuous heparin infusion		
Yes, % (n)		23.7 (89)
No, % (n)		75.9 (286)
Laboratory exams at first RRT indication		
Creatinine (mg/dl)	0.6–1.3	4.05 (2.90–5.60)
Urea (mg/dl)	16–40	152.8 (98.5–211.0)
Potassium (mEq/l)	3.5–5.1	4.8 (4.2–5.5)
Bicarbonate (mEq/l)	22–27	22.1 (19.0–25.9)
Exams during the RRT period		
Creatinine (mg/dl)	0.6–1.3	3.63 (2.12–5.10)
Urea (mg/dl)	16–40	126.5 (88.0–174.1)
Potassium (mEq/l)	3.5–5.1	4.7 (4.1–5.4)
Bicarbonate (mEq/l)	22–27	23.0 (19.6–26.0)
RRT method		
Intermittent, % (n)		56.5 (212)
SLED, % (n)		8.3 (31)
Continuous, % (n)		18.7 (70)
Combination of methods, % (n)		16.5 (62)
Time on RRT (days)		6 (2–15)
Outcomes		
Total length of stay (days)		19 (11–30)
Death, % (n)		72.5 (272)
Discharge without RRT, % (n)		77.7 (80/103)
Discharge dependent on RRT, % (n)		22.3 (23/103)

Data are presented as the mean ± SD, the median and interquartile range (p25-p75), or a percentage. SLED, sustained low-efficiency dialysis.

In the univariate analysis, nonsurvivors exhibited a shorter hospital stay than survivors (15 [9–23] vs. 36 [22–47] days, p < 0.001); a higher frequency of circulatory dysfunction (89.7% vs. 73.8%, p < 0.001), pulmonary dysfunction (82.0% vs. 69.9%, p = 0.01), and liver dysfunction (19.1% vs. 6.3%, p = 0.004); and a higher frequency of corticosteroid use (65.7% vs. 46.6%, p = 0.001).

Nonsurvivors had higher serum potassium levels (4.9 [4.3–5.6] vs. 4.5 [3.9–5.2] mEq/l, p = 0.003) on the day of the first RRT indication. In addition, nonsurvivors had higher simple medians of creatinine (3.72 [2.30–5.25] vs. 3.20 [1.80–4, 82] mg/dl, p = 0.04), urea (131.5 [91.0–180.4] vs. 108.4 [79.5–159.2] mg/dl, p = 0.03), and potassium (4.8 [4.2–5.5] vs. 4.3 [3.8–4.9] mEq/l, p < 0.001), and lower serum bicarbonate (22.5 [19, 1–25.6] vs. 23.8 [20.6–27.4] mEq/l, p = 0.006) during the RRT period (Table 3).

**Table 3:** Comparison between surviving and nonsurviving patients.

	Survivors (N = 103)	No survivors (N = 272)	P value
Demographics			
Age, years	62.5 (54.0–74.3)	64.9 (55.4–74.2)	0.38
Male sex, % (n)	68.9 (71)	68.8 (187)	1.00
White ethnicity, % (n)	56.3 (40)	65.8 (106)	0.16
Smoking, % (n)	18.4 (19)	18.1 (49)	0.95
Comorbidities			
Hypertension, % (n)	69.9 (72)	67.3 (183)	0.63
Diabetes mellitus, % (n)	49.5 (51)	43.8 (119)	0.32
Obesity, % (n)	31.0 (26)	43.3 (90)	0.05
Heart failure, % (n)	8.7 (9)	11.0 (30)	0.52
Coronary insufficiency, % (n)	13.6 (14)	16.5 (45)	0.48
CKD, % (n)	15.5 (16)	19.9 (54)	0.33
COPD, % (n)	6.8 (7)	9.2 (25)	0.46
Chronic liver disease, % (n)	2.9 (3)	1.8 (5)	0.54
Neoplasia, % (n)	4.9 (5)	3.0 (8)	0.38
Participating institution, public hospital, % (n)	59.2 (61)	61.0 (166)	0.75
Mean arterial pressure at hospital admission (mmHg)	94 ± 19	90 ± 18	0.07
Oxygen saturation at hospital admission (%)	91 (88–95)	92 (88–95)	0.86
Time to symptom onset (days)	5 (3–7)	5 (3–8)	0.28
Hospital admission exams			
Hemoglobin (g/dl) (N.R. 12.0–16.0)	13.3 (11.9–14.3)	13.2 (11.8–14.5)	0.51
Total leukocytes (n/mm^3^) (N.R. 4,000–8,000)	8.425 (5.658–11.600)	8020 (5475–11040)	0.26
Total lymphocytes (n/mm^3^) (N.R. 1,000–3,900)	909 (699–1348)	995 (697–1516)	0.37
Platelets (10^3^/mm^3^) (N.R. 150,000–400,000)	186 (143.5–260.1)	177 (141–225)	0.09
Serum creatinine (mg/dl) (N.R. 0.6–1.3))	1.20 (0.94–1.75)	1.14 (0.89–1.80)	0.21
D-dimer (ng/ml) (N.R. < 0.5)	1.30 (0.64–8.94)	1.83 (0.78–9.2)	0.25
C-reactive protein (mg/dl) (N.R. < 0.1)	16.8 (10–5–28.2)	14.9 (7.0–22.9)	0.07
SAPS 3	52 (44–61)	54 (47–67)	0.13
Severe involvement on tomography, % (n)	40.8 (42)	39.3 (107)	0.46
**Mechanical ventilation**	**80.6 (83)**	**91.5 (249)**	**0.003**
Organ dysfunctions			
** Circulatory, % (n)**	**73.8 (76)**	**89.7 (244)**	**< 0.001**
** Pulmonary, % (n)**	**69.9 (72)**	**82.0 (223)**	**0.011**
Coagulopathy, % (n)	23.3 (24)	33.3 (90)	0.06
** Liver, % (n)**	**6.3 (6)**	**19.1 (44)**	**0.004**
Medications			
** Vasoactive drug, % (n)**	**73.8 (76)**	**89.7 (244)**	**< 0.001**
** Vasopressin, % (n)**	**14.6 (13)**	**52.1 (124)**	**< 0.001**
** Noradrenaline, % (n)**	**72.8 (75)**	**88.6 (241)**	**< 0.001**
** Dobutamine, % (n)**	**7.9 (6)**	**18.7 (38)**	**0.03**
Azithromycin + hydroxychloroquine, % (n)	41.7 (43)	36.8 (99)	0.47
** Corticosteroids, % (n)**	**46.6 (48)**	**65.7 (178)**	**0.001**
Continuous heparin infusion, % (n)	24.5 (25)	24.0 (64)	0.91
Laboratory exams at first RRT indication			
Creatinine (mg/dl) (N.R. 0.6–1.3)	4.00 (2.90–5.52)	4.10 (2.90–5.60)	0.78
Urea (mg/dl) (N.R. 16–40)	140.0 (86.5–212.5)	157.5 (100.2–211.0)	0.18
**Potassium (mEq/l)** (N.R. 3.5–5.1)	**4.5 (3.9–5.2)**	**4.9 (4.3–5.6)**	**0.003**
Bicarbonate (mEq/l) (N.R. 22–27)	23.0 (18.9–26.0)	22.0 (19.0–25.3)	0.31
Laboratory values during the RRT period			
**Creatinine (mg/dl)** (N.R. 0.6–1.3)	**3.20 (1.80–4.82)**	**3.72 (2.30–5.25)**	**0.04**
**Urea (mg/dl)** (N.R. 16–40)	**108.4 (79.5–159.2)**	**131.5 (91.0–180.4)**	**0.03**
**Potassium (mEq/l)** (N.R. 3.5–5.1)	**4.3 (3.8–4.9)**	**4.8 (4.2–5.5)**	**< 0.001**
**Bicarbonate (mEq/l)** (N.R. 22–27)	**23.8 (20.6–27.4)**	**22.5 (19.1–25.6)**	**0.006**
RRT method			
Intermittent, % (n)	53.4 (55)	57.7 (157)	0.45
SLED, % (n)	9.7 (10)	7.7 (21)	0.53
Continuous, % (n)	18.4 (19)	18.8 (51)	0.94
Combination of methods, % (n)	18.4 (19)	15.8 943)	0.54
**Time on RRT (days)**	**15 (7–24)**	**4 (2–11)**	**< 0.001**
**Length of hospital stay (days)**	**36 (22–47)**	**15 (9–23)**	**< 0.001**

Data are presented as the mean ± SD, the median and interquartile range (p25-p75), or a percentage. SLED, sustained low-efficiency dialysis, N.R., normal range.

Among the survivors, those discharged while dependent on RRT exhibited a higher frequency of obesity (52.9% vs. 25.4%, p = 0.03) and higher use of isolated IHD (82.6% vs. 57.5%, p = 0.03) based on the univariate analysis. They showed higher serum creatinine levels (2.95 [1.32–6.97] vs. 1.07 [0.90–1.53] mg/dl, p < 0.001) and lower total leukocyte counts (7,275 [5,25–9,185] vs. 13,050 [6,105–13,242]/mm^3^, p = 0.04) at admission. (Table 4).

**Table 4:** Comparison between patients discharged while dependent and not dependent on kidney replacement therapy.

	Discharged without dependence on RRT (N = 80)	Discharged while dependent on RRT (N = 23)	P value
Demographics			
Age, years	62.5 (54.1–74.2)	62.3 (53.4–74.9)	0.94
Male sex, % (n)	70.0 (56)	65.2 (15)	0.66
Comorbidities			
Hypertension, % (n)	71.3 (57)	65.2 (15)	0.58
Diabetes mellitus, % (n)	46.3 (37)	60.9 (14)	0.22
** Obesity, % (n)**	**25.4 (17)**	**52.9 (9)**	**0.03**
Heart failure, % (n)	10.0 (8)	4.3 (1)	0.40
Coronary insufficiency, % (n)	12.5 (10)	17.4 (4)	0.57
CKD, % (n)	17.5 (14)	8.7 (2)	0.28
Hospital admission exams			
Hemoglobin (g/dl) (N.R. 12.0–16.0)	13.0 (11.9–14.3)	13.8 (11.9–15.3)	0.25
**Total leukocytes (n/mm**^**3**^**)** (N.R. 4,000–8,000)	**13.050 (6.105–13.242)**	**7.275 (5.025–9.185)**	**0.04**
Total lymphocytes (n/mm^3^) (N.R. 1,000–3,900)	931 (699–1305)	852 (699–1495)	0.79
Platelets (10^3^/mm^3^) (N.R. 150,000–400,000)	193 (151–272)	158 (120–245)	0.11
**Serum creatinine (mg/dl)** (N.R. 0.6–1.3)	**1.07 (0.9–1.53)**	**2.95 (1.32–6.97)**	**< 0.001**
SAPS 3	51 (44–61)	56 (42–69)	0.70
Severe pulmonary involvement on tomography, % (n)	45.2 (33)	42.9 (9)	0.81
Number of organ dysfunctions	2 (1–2)	2 (1–2)	0.36
Laboratory exams at first RRT indication			
Creatinine (mg/dl) (N.R. 0.6–1.3)	4.00 (2.85–5.44)	4.6 (4.2–5.32)	0.28
Urea (mg/dl) (N.R. 16–40)	130 (83–198)	173 (120–243)	0.05
Potassium (mEq/l) (N.R. 3.5–5.1)	4.5 (3.9–5.2)	4.8 (4.2–5.3)	0.09
Bicarbonate (mEq/l) (N.R. 22–27)	23.0 (19.7–26.6)	21.2 (18.8–25.1)	0.14
Laboratory values during the RRT period			
Creatinine (mg/dl) (N.R. 0.6–1.3)	2.73 (1.80–4.59)	3.85 (2.47–5.17)	0.10
Urea (mg/dl) (N.R. 16–40)	110 (79–154)	99 (77–165)	0.76
Potassium (mEq/l) (N.R. 3.5–5.1)	4.3 (3.8–4.8)	4.7 (3.9–5.2)	0.13
Bicarbonate (mEq/l) (N.R. 22–27)	24.0 (20.8–27.6)	23.4 (19.3–26.3)	0.27
RRT method			
** Intermittent, % (n)**	**57.5 (46)**	**82.6 (19)**	**0.03**
SLED, % (n)	7.5 (6)	0.0 (0)	0.33
Continuous, % (n)	11.3 (9)	4.3 (1)	0.45
Combination of methods, % (n)	23.8 (19)	13.0 (3)	0.39
Time on RRT (days)	15 (6–23)	15 (7–36)	0.23
Length of hospital stay (days)	37 (24–47)	26 (16–45)	0.06

Data are presented as the medians and interquartile ranges (p25-p75) or as percentages; RRT: Kidney replacement therapy; CKD: Chronic kidney disease. SLED, sustained low-efficiency dialysis; N.R., normal range.

The independent factors associated with death in the multiple logistic regression analysis were the number of organ dysfunctions during hospitalization and efficient RRT (Table 5).

**Table 5:** Multivariate analysis of factors related to death.

Variable	Adjusted OR (CI 95%)	P value
Number of organ dysfunctions	1.52 (1.11–2.09)	0.009
Efficient RRT	0.41 (0.25–0.69)	< 0.001

Multiple logistic regression with variables adjusted for type of hospital, age, sex, smoking, number of comorbidities, lower tercile of platelets and higher creatinine at admission, use of vasoactive drugs, mechanical ventilation, and serum measurements (potassium < 5 mEq/ml, bicarbonate > 22 mEq/ml, urea > 150 mg/dl, and upper tercile of creatinine). OR: Odds ratio; RRT: Kidney replacement therapy.

## Discussion

The results of this multicenter study in the metropolitan region of the megalopolis of São Paulo indicated that AKI-RRT associated with COVID-19 has high lethality and occurs mainly in patients with a high burden of comorbidities and other organ dysfunctions related to the critical state. A higher number of organ dysfunctions increased and efficient RRT decreased the OR for death in the multiple regression analysis. More than one-fifth of the survivors were discharged from the hospital while dependent on RRT, an outcome associated with obesity and a higher creatinine level at hospital admission. To date, this is the largest report related to the epidemiological profile and outcomes of critically ill patients with AKI-RRT and COVID-19 in the Southern Hemisphere.

In our study, the lethality of critically ill patients with COVID-19 and AKI-RRT was similar to that reported in other multicenter international studies (63–79%) [12–15,20]. Unfortunately, few studies have reported the SAPS score (severity), impeding opportunities to compare populations. Nevertheless, the percentages of patients who required mechanical ventilation and vasoactive drugs reported in these studies were similar to those found in the present study (79–97% and 51–92%, respectively). The lower lethality (63%) found in the cohort in the study by Gupta et al. may be partly explained by the lower severity of disease in the patients (79% and 51% required mechanical ventilation and vasopressor use, respectively) [13]. The independent risk factors for death reported in other studies were age, obesity, COPD, coronary artery disease, neoplasia, oliguria on the day of the first indication for RRT, and the need for two or more vasopressors [13,15,16]. In our study, the presence of pulmonary, circulatory, and liver dysfunctions alone and in combination was associated with death.

The present study showed that patients with higher potassium and urea and lower bicarbonate levels during the RRT period had higher mortality rates. These findings may suggest that an insufficient RRT dose affected patient outcomes. Another possible explanation is that more severe disease and hemodynamic instability hindered adequate metabolic control. In fact, in the present study, patients who died received fewer days of RRT and had shorter hospital stays. In previous studies, information on laboratory tests at the beginning of RRT and metabolic control for patients with AKI-RRT and COVID-19 has been scarce or absent. Oliguria at RRT initiation was reported as an independent risk factor for death in patients with these conditions, potentially indicating more severe AKI as well as a delay in the indication for RRT [13]. Unfortunately, the present study was unable to obtain information on the participants’ urine output and fluid balance.

The RRT method was likely not directly related to mortality in the present study or in other studies involving AKI in patients with COVID-19 [15,19,20]. The percentage of patients who underwent continuous RRT in this study was lower than those in other multicenter studies (46–98%) [13,15,16,20], reinforcing the hypothesis that factors other than the RRT method determine the outcomes of critically ill patients with AKI-RRT. In Brazil, continuous RRT is the modality of choice in a few private hospitals and university centers [21,22]. No patient participating in this study was treated with PD, which is consistent with previous reports on RRT methods used more frequently in Latin America. However, studies have shown that PD can be successfully used in adults with AKI, including COVID-19-associated AKI [23–26].

As in our study, other authors reported that COVID-19-associated AKI-RRT occurred more frequently in men (68–83%) and in patients with a high burden of comorbidities (62–70 years of age; HTN, 62–71%; diabetes, 40–53%; obesity, 41–45%; CKD, 26–35%; and coronary artery disease, 13–16%) [12–15,20]. Although the Kidney Disease Improving Global Outcomes guideline [27] includes female sex on the list of susceptibilities for AKI, a higher prevalence of males with severe forms of AKI has been described for patients with and without COVID-19 [2,28–31]. The present study included only patients affected by the first wave of COVID-19 and showed a high burden of comorbidities in this cohort of critically ill patients treated with RRT. During the second wave, although the mean age of affected patients was lower, high percentages of HTN, diabetes, CKD, and HF continued to be reported in individuals with severe forms of the disease [32,33].

In previous studies, among survivors of AKI-RRT associated with COVID-19, the percentage discharged from the hospital while dependent on RRT varied between 30–38% [12–15]. The relatively lower rate of requiring RRT at discharge observed in our study (22%) may be associated with the lower proportion of patients with CKD (19%). In fact, the presence of CKD in patients with and without COVID-19 is the main risk factor for RRT dependence after an AKI-RRT event [14,31].

The limitations of this study are the retrospective design, the lack of data from all patients who met the inclusion criteria, and the lack of information on kidney function recovery after hospital discharge. In addition, considering the regional differences in Brazil and the fact that the global mortality of critically ill patients has a strong association with national income [34], the results presented here may not represent Brazilian ICUs or those in the Southern Hemisphere. In fact, a recent Brazilian study conducted in public hospitals with limited resources showed a mortality rate of 93% for patients with COVID-19 and severe AKI [35].

## Conclusion

COVID-19-associated AKI-RRT occurred in patients with an elevated burden of comorbidities and showed high lethality. The number of organ dysfunctions during hospitalization and the efficiency of RRT were independent factors associated with mortality. A significant portion of the survivors were discharged without kidney function recovery.

## Supporting information

S1 File (XLSX)
